# A Group Comparison Test under Uncertain Group Membership

**DOI:** 10.1007/s11336-021-09794-x

**Published:** 2021-08-26

**Authors:** Tobias A. Bauer, Alexandro Folster, Tina Braun, Timo von Oertzen

**Affiliations:** 1 University of the BUNDESWEHR, MUNICH, Munich, Neubiberg Germany; 2 Max Planck Institute for Human Development, Berlin, Germany

**Keywords:** *t* test, group uncertainty

## Abstract

An overwhelming majority of articles in psychology compare means, often between multiple groups. However, sometimes we do not know the exact group membership, but only a probability to be in one of the groups. Such information may come from classifiers trained on other datasets, prevalence of group memberships for some parts of the sample, multi-level situations where the group membership is only known as a ratio in an upper level, or expert ratings (e.g., whether a person has a pathological condition or not). We present a simple method that allows to compare group means in the absence of exact knowledge about group membership and investigate the loss of information depending on the probability values theoretically and in a large-scale simulation.

Mean comparisons with *t*-tests are probably one of the most commonly used methods in behavioral sciences. The classical independent *t*-test, used to compare the mean of two samples, requires one normally distributed dependent variable and uses a group membership variable as the independent variable (Student 1908).

However, there are multiple research situations where the group membership itself is not available or not reliably available. Nevertheless, a probability to be in one of the two groups may be available instead. The information about the probability may come from different sources; for example, (1) expert rating, (2) known probabilities from full-population or larger datasets, (3) automatic probability ratings, and (4) deliberate randomization of group memberships to protect sensitive data of participants. We will give short conceptual illustrations of these four cases.

Assume a researcher wants to investigate within a mining company whether workers that live in the towns next to the mines are more efficient. If the miners cannot be asked individually about the place they live, a foreman of the mine may still be able to make this call, with certainty for some, but only with a certain error probability for others. So, although no certain group memberships are known, a probability is available. As an example for the second setting, assume that workers come from three different shifts, where group membership could be assessed for two shifts but not for the night shift. However, the records of the company give the overall probability for miners in the night shift to live close by; so again, although no certain group membership is available, the overall prevalence of miners living close by can be used as the probability for individual miners in the sample of the night shift. Thirdly, even if the general records do not include the address, it may contain other variables unrelated to work performance but allowing predictions whether someone lives close by, for example, tax confirmations which are required more likely for those with longer commutes, but not directly related to work performance. These predictions could for example be done by a classifier (Borgelt et al. 2009, Berthold et al. 2010, Platt 1999, Ho 1995, Rusk 2016) with a quantifiable success probability. Fourthly, assume living in a specific township is sensitive information that miners may not be willing to share openly. Then, the information whether a miner lives in the close-by town may be asked using a forced response technique (a variant of the randomized response technique by Boruch 1971), where participants roll a die covertly before they answer the questions, and give the opposite answer if they rolled a six. In this case, the probability to be in the group named by the participant is five sixth.

Frequently, data in which the group variable is completely or partially missing is considered unsuitable for a *t*-test, or at least those participants with uncertain group membership are eliminated from the dataset in a listwise-delete handling of missingness (e.g., deWolff et al. 2019, Harkonen et al. 2018, Moise 2019). Although missing certain information, as, i.e., group membership, already collected data still includes information contributing to the research question. Listwise-deletion of several participants or deletion of whole datasets results in the waste of already gathered information and, therefore, causes the overall research to be less economical regarding time spent by participant and experimenter but also monetary resources. The current article suggests a method that enables the researcher to use incomplete datasets by calculating *t*-values with grouping variables given as probabilities for group membership instead of categorical grouping variables. It assumes the usual *t*-test assumptions of (1) two normally distributed groups with (2) equal variance, and in addition that the group membership probability are (3) given in the dataset and (4) independent of the dependent variable within each group. We suggest a statistic for the group mean difference with a known distribution under any true group membership, which can be used in a frequentist test as well as in a Bayesian estimation procedure (Jackman 2009).

The problem can be viewed as a missingness problem where the group membership is missing in some or all participants. An example is the situation described above, where we do not know the place of residence for all miners. Yet, we know the probability of them living next to the mine. Classical treatment of missingness will not make use of the probabilities and therefore ignore an important part of the data, necessarily leading to a bias. This is obvious with listwise deletion methods (Graham et al. 2003), which in the current case is identical to a pairwise deletion. However, the same is also true for multiple imputation (Royston 2004) or full information maximum likelihood (Enders and Bandalos 2001) approaches.

The problem described here may be seen as a special case of latent class modeling (LCA; Vermunt and Magidson 2004) with a continuous distal outcome (Bakk and Vermunt 2015) in which the class probabilities of each participant are not estimated but set to the true values given in the dataset. The optimization in such models can for example be done by a 3-step approach (BCH; Bakk and Vermunt 2004; 2010). LCA has been introduced primarily to *detect* the probability for each participant to be in a specific class. The method suggested here works in the opposite direction, assuming that the probabilities to be in either class are already known. This allows both to not loose power that gets diverted into estimating the class probabilities and at the same time to use a considerably simpler method than a full LCA.

In fact, the algorithm in the current article is simpler in comparison: A statistic is calculated, which asymptotically approaches the group mean difference. The distribution of this statistic can be approximated, allowing for the computation of confidence intervals and a *t*-distributed statistic.

In the following, we will outline the mathematical background of the method and provide equations for the difference statistic and the corresponding *t*-value, both in a broader mathematical description and a quick lookup-table for the applied user. We will then show a simulation to demonstrate that the method works, and depict its power under different effect sizes and information about the group membership. In addition, two concrete examples will be given how the test could be used. These will be based on the openly available PISA 2018 data (OECD 2018). The article concludes with a discussion of the limitations and applications of the method.

## Mathematical Derivation

The current section introduces the mathematical background and provides proofs for the correctness of the uncertain group *t*-test.

Classical Student’s *t*-test requires dichotomous variables to code the group identity, while the current article introduces a simple method to compute a statistic for the mean difference between two groups when only a probability to be in either group is given for every participant. The classical *t*-test is a special case of this where each probability is either 0% or 100%. We will first provide the statistics with a frequentist test and then introduce a way to use the statistics for Bayesian estimation.

### Frequentist Comparison of Uncertain Groups

Assume a dataset consisting of *N* data vectors from two groups labeled 1 and 2. Each data vector has two values $(x_i, p_i)$ where $0 \le p_i \le 1$ is the probability that person *i* is in Group 1. $x_i$ is distributed by a 2-mixture of Gaussians independent of each other and of $p_i$ with variances $\sigma _1^2$ and $\sigma _2^2$ and means $\mu _1$ and $\mu _2$ within each group. Every single data vector is thus distributed as a mixture of Gaussians. Data vectors are assumed to be independent. We aim at defining a test under the assumption that $\sigma _1^2 = \sigma _2^2$ to test whether $\mu _1 = \mu _2$.

We write ${\overline{p}}$ for the average $\frac{1}{N} \sum _{i=1}^N p_i$ of $p_i$ and ${\overline{x}}$ for the average $\frac{1}{N} \sum _{i=1}^N x_i$ of $x_i$. We will furthermore write $\mathbb {V}(p) = \frac{1}{N} \sum _{i=1}^N (p_i - {\overline{p}})^2$ for the sample variance of $p_i$. Let ${\hat{\sigma }}$ be a suitable unbiased estimate of the standard deviation under the assumption that both distributions are equal.

The following theorem is central for the method and provides a statistic for the mean difference and a *t*-distributed test statistic. The proof is not required for the application of the test, readers who are mainly interested in the application may continue at section “Practical Computations.”

#### Theorem 1

Let

$$\begin{aligned} d = \frac{\sum _{i=1}^N (p_i - {\overline{p}}) x_i}{N \mathbb {V}(p)} \end{aligned}$$

and

$$\begin{aligned} t = d \cdot \frac{\sqrt{N \mathbb {V}(p)}}{{{\hat{\sigma }}}}. \end{aligned}$$

Then, *d* is an unbiased estimate of $\mu _1 - \mu _2$ with variance

$$\begin{aligned} \mathbb {V}(d) = \frac{ \sum _{i=1}^N (p_i - {\overline{p}})^2 \left[ p_i (1-p_i) (\mu _1-\mu _2)^2 + p_i \sigma _1^2 + (1-p_i) \sigma _2^2\right] }{N^2 \mathbb {V}(p)^2} \end{aligned}$$

and *t* is *t*-distributed with $N-1$ degrees of freedom under the null hypothesis that both distributions are equal.

#### Proof

Let

$$\begin{aligned} z = \sum _{i=1}^N (p_i - {\overline{p}}) x_i \end{aligned}$$

be the numerator of *d*. The expectation of *z* is

1 $$\begin{aligned} \mathbb {E}(z)= & {} \sum _{i=1}^N (p_i - {\overline{p}}) \mathbb {E}(x_i) \end{aligned}$$

2 $$\begin{aligned}= & {} \sum _{i=1}^N (p_i - {\overline{p}}) (p_i (\mu _1 - \mu _2) + \mu _2) \end{aligned}$$

3 $$\begin{aligned}= & {} \sum _{i=1}^N (p_i - {\overline{p}}) p_i (\mu _1 - \mu _2) + \mu _2 \sum _{i=1}^N (p_i - \overline{p_i}) \end{aligned}$$

4 $$\begin{aligned}= & {} \sum _{i=1}^N (p_i - {\overline{p}}) p_i (\mu _1 - \mu _2) \end{aligned}$$

5 $$\begin{aligned}= & {} (\mu _1 - \mu _2) N \left( \frac{1}{N} \sum _{i=1}^N p_i^2 - \left( \frac{1}{N} \sum _{i=1}^N p_i \right) ^2 \right) \end{aligned}$$

6 $$\begin{aligned}= & {} (\mu _1 - \mu _2) N \mathbb {V}(p) \end{aligned}$$

so that $\mathbb {E}(d) = (\mu _1 - \mu _2)$. To compute the variance of *z*, we first start by computing the variance of $x_i$:

7 $$\begin{aligned} \mathbb {V}(x_i)= & {} \mathbb {E}(x_i^2) - \mathbb {E}(x_i)^2 \end{aligned}$$

8 $$\begin{aligned}= & {} p_i (\sigma _1^2 + \mu _1^2) + (1-p_i) (\sigma _2^2 + \mu _2^2) - (p_i (\mu _1-\mu _2) + \mu _2)^2 \end{aligned}$$

9 $$\begin{aligned}= & {} p_i (\sigma _1^2 + \mu _1^2 - \sigma _2^2 - \mu _2^2) + (\sigma _2^2+\mu _2^2) - p_i^2 (\mu _1-\mu _2)^2 - 2 p_i (\mu _1-\mu _2) \mu _2 - \mu _2^2 \end{aligned}$$

10 $$\begin{aligned}= & {} -(\mu _1-\mu _2)^2 p_i^2 + p_i (\sigma _1^2 - \sigma _2^2 + \mu _1^2 - 2 \mu _1 \mu _2 + \mu _2^2) - \sigma _2^2 \end{aligned}$$

11 $$\begin{aligned}= & {} p_i (1-p_i) (\mu _1-\mu _2)^2 + p_i \sigma _1^2 + (1-p_i) \sigma _2^2. \end{aligned}$$

Therefore, the variance of *z* is

12 $$\begin{aligned} \mathbb {V}(z)= & {} \sum _{i=1}^N (p_i - {\overline{p}})^2 \mathbb {V}(x_i) \end{aligned}$$

13 $$\begin{aligned}= & {} \sum _{i=1}^N (p_i - {\overline{p}})^2 \left[ p_i (1-p_i) (\mu _1-\mu _2)^2 + p_i \sigma _1^2 + (1-p_i) \sigma _2^2\right] . \end{aligned}$$

The variance of *d* is then

14 $$\begin{aligned} \mathbb {V}(d)= & {} \frac{\mathbb {V}(z)}{(N \mathbb {V}(p))^2} \end{aligned}$$

15 $$\begin{aligned}= & {} \frac{ \sum _{i=1}^N (p_i - {\overline{p}})^2 \left[ p_i (1-p_i) (\mu _1-\mu _2)^2 + p_i \sigma _1^2 + (1-p_i) \sigma _2^2\right] }{N^2 \mathbb {V}(p)^2} \end{aligned}$$

which proves the first statement.

If we assume that the two distributions are equal, this simplifies to

16 $$\begin{aligned} \mathbb {V}(d | \mu _1=\mu _2, \sigma _1 = \sigma _2)= & {} \frac{ \sum _{i=1}^N (p_i - {\overline{p}})^2 \sigma ^2}{N^2 \mathbb {V}(p)^2} \end{aligned}$$

17 $$\begin{aligned}= & {} \frac{ \sigma ^2 \sum _{i=1}^N (p_i - {\overline{p}})^2}{N^2 \mathbb {V}(p)^2} \end{aligned}$$

18 $$\begin{aligned}= & {} \frac{ \sigma ^2 }{N \mathbb {V}(p)} \end{aligned}$$

and, hence, $\mathbb {V}(t) = 1$, while still $\mathbb {E}(t) = 0$, which proves the second statement. $\square$

To allow for some intuition, note that *z* can also be written as

19 $$\begin{aligned} z= & {} \sum _{i=1}^N (p_i - {\overline{p}}) x_i = \sum _{i=1}^N p_i x_i - \frac{1}{N} \left( \sum _{i=1}^N p_i\right) \left( \sum _{i=1}^N x_i\right) \end{aligned}$$

20 $$\begin{aligned}= & {} \sum _{i=1}^N p_i (x_i - {\overline{x}}). \end{aligned}$$

In this representation, we can see that if all $p_i$ are equal, *z* will always be zero, and *d* undefined. If all $p_i$ are either 0 or 1, then *d* will be the difference of the two group averages in the sample, and *t* will be the classical *t*-test value.

Note further that $z = {\hat{cov}}(x,p)$ where ${\hat{cov}}$ is the population covariance of *x* and *p* from the data. With this, *d* can be written as

$$\begin{aligned} d = \frac{{\hat{cov}}(x,p)}{\mathbb {V}(p)} \end{aligned}$$

and

$$\begin{aligned} t = {\hat{cor}}(x,p) \cdot \sqrt{N-1} \end{aligned}$$

where ${\hat{cor}}$ is the population correlation.

The following lemma allows us to isolate the means of both groups separately if necessary:

#### Lemma 2

$$\begin{aligned} \mathbb {E}\left( {\overline{x}} - d {\overline{p}} \right) = \mu _2 \end{aligned}$$

and accordingly

$$\begin{aligned} \mathbb {E}\left( {\overline{x}} + d (1-{\overline{p}}) \right) = \mu _1 \end{aligned}$$

#### Proof

21 $$\begin{aligned} \mathbb {E}\left( {\overline{x}} - d {\overline{p}} \right)= & {} \frac{1}{N} \sum _{i=1}^N \mathbb {E}(x_i) - {\overline{p}}\mathbb {E}(d) \end{aligned}$$

22 $$\begin{aligned}= & {} \frac{1}{N} \sum _{i=1}^N \left( p_i (\mu _1-\mu _2) + \mu _2\right) - {\overline{p}} (\mu _1-\mu _2) \end{aligned}$$

23 $$\begin{aligned}= & {} (\mu _1-\mu _2) {\overline{p}} + \frac{1}{N} N \mu _2 - {\overline{p}} (\mu _1 - \mu _2) \end{aligned}$$

24 $$\begin{aligned}= & {} \mu _2 \end{aligned}$$

and the second statement follows simply from

$$\begin{aligned} \mu _1 = d + \mu _2 = d + {\overline{x}} - d {\overline{p}} = {\overline{x}} + d (1-{\overline{p}}). \end{aligned}$$

$$\square $$

We can find the second moment of both groups separately by first computing an expression of the difference of second moments and then using $\sum _{i=1}^N x_i^2$ to isolate both separate second moments analogously to Lemma 2.

#### Theorem 3

Let

$$\begin{aligned} z_2 = \sum _{i=1}^N (p_i - {\overline{p}}) x_i^2. \end{aligned}$$

Then,

$$\begin{aligned} \mathbb {E}\left( \frac{z_2}{N \mathbb {V}(p)}\right) = (\sigma _1^2 + \mu _1^2 - \sigma _2^2 - \mu _2^2) \end{aligned}$$

and

25 $$\begin{aligned} \mathbb {E}\left( \frac{\sum _{i=1}^N x_i^2}{N} - \frac{z_2 {\overline{p}}}{N \mathbb {V}(p)}\right)= & {} (\sigma _2^2 + \mu _2^2) \end{aligned}$$

26 $$\begin{aligned} \mathbb {E}\left( \frac{\sum _{i=1}^N x_i^2}{N} - \frac{z_2 (1-{\overline{p}})}{N \mathbb {V}(p)}\right)= & {} (\sigma _1^2 + \mu _1^2). \end{aligned}$$

#### Proof

The second moment of *x* is the linear mixture of the second moments from both mixture distributions,

$$\begin{aligned} \mathbb {E}(x_i^2) = p_i (\sigma _1^2 + \mu _1^2) + (1-p_i) (\sigma _2^2 + \mu _2^2). \end{aligned}$$

Using this in the identical computation as in the proof to Theorem 1, we get

$$\begin{aligned} \mathbb {E}(z_2) = (\sigma _1^2 + \mu _1^2 - \sigma _2^2 - \mu _2^2) N \mathbb {V}(p) \end{aligned}$$

which proves the first statement.

The second and third statements follow in analogy to the proof of Lemma 2. $\square$

The estimate of the second moments as given above is unbiased. When using the mean estimator $\mu _j$ for one group to isolate the variance $\sigma _j^2$,

$$\begin{aligned} {\hat{\sigma _j}}^2 = \frac{\sum _{i=1}^N x_i^2}{N} - \frac{z_2 {\overline{p}}}{N \mathbb {V}(p)} - {\hat{\mu }}_2^2 \end{aligned}$$

the estimate is biased by a factor that depends on both group variances. Assuming these two are equal, the bias factor is $\frac{N-1}{N}$.

If we assume for the test that both group variances are equal, we get a unique variance estimate as described by the following lemma.

#### Lemma 4

$$\begin{aligned} {\hat{\sigma }}^2 = \frac{1}{N-2} \left( \left( \sum _{i=1}^N (x_i - {\overline{x}})^2\right) - N {\overline{p}} (1-{\overline{p}}) d^2 \right) . \end{aligned}$$

${\hat{\sigma }}^2$, therefore, is an unbiased estimate for the variance. If both means are equal, the latter term is zero. If we assume that this equality is known, one degree of freedom is removed from the equation. Hence, under this assumption,

$$\begin{aligned} {\hat{\sigma }}^2 = \frac{1}{N-1} \sum _{i=1}^N (x_i - {\overline{x}})^2 \end{aligned}$$

is an unbiased estimate for the common variance.

#### Proof

We prove the first statement assuming we know both means, and then correct for the two degrees of freedom. We find that

27 $$\begin{aligned} \mathbb {E}\left( \sum _{i=1}^N (x_i - {\overline{x}})^2\right)= & {} \sum _{i=1}^N \mathbb {E}(x_i^2) - N \mu ^2 \end{aligned}$$

28 $$\begin{aligned}= & {} \sum _{i=1}^N p_i (\sigma ^2+\mu _1^2) + (1-p_i) (\sigma ^2+\mu _2^2) \end{aligned}$$

29 $$\begin{aligned}&- \frac{1}{N} \left( \sum _{i=1}^n \mu _1 p_i + \sum _{i=1}^n \mu _2 (1-p_i)\right) ^2 \end{aligned}$$

30 $$\begin{aligned}= & {} \sum _{i=1}^N \sigma ^2 + p_i (\mu _1^2 - \mu _2^2) + \mu _2^2 - \frac{1}{N} \left( N {\overline{p}} \mu _1 + N (1-{\overline{p}}) \mu _2 \right) ^2 \end{aligned}$$

31 $$\begin{aligned}= & {} N \left[ \sigma ^2 + {\overline{p}} (\mu _1^2 - \mu _2^2) + \mu _2^2 - {\overline{p}}^2 (\mu _1-\mu _2)^2 - 2 {\overline{p}} (\mu _1-\mu _2) \mu _2 - \mu _2^2 \right] \end{aligned}$$

32 $$\begin{aligned}= & {} N \left[ \sigma ^2 + {\overline{p}} (\mu _1^2 - \mu _2^2) - {\overline{p}}^2 (\mu _1 - \mu _2)^2 - 2 {\overline{p}} (\mu _1 - \mu _2) \mu _2 \right] \end{aligned}$$

33 $$\begin{aligned}= & {} N \left[ \sigma ^2 + \mu _1^2 ({\overline{p}} - {\overline{p}}^2) + \mu _2^2 ({\overline{p}}- {\overline{p}}^2) + \mu _1 \mu _2 (2 {\overline{p}}^2 - 2 {\overline{p}})\right] \end{aligned}$$

34 $$\begin{aligned}= & {} N \left[ \sigma ^2 + {\overline{p}} (1-{\overline{p}}) \left( \mu _1^2 + \mu _2^2 - 2 \mu _1\mu _2\right) \right] \end{aligned}$$

35 $$\begin{aligned}= & {} N \left[ \sigma ^2 + {\overline{p}} (1-{\overline{p}}) (\mu _1 - \mu _2)^2 \right] \end{aligned}$$

so the first statement is shown. If we assume $\mu _1 = \mu _2$ in addition, we are back to the classical situation in which all variables have the same variance and mean, so the second statement is well known. $\square$

### Bayesian use of Uncertain Groups

As before, we will assume $\sigma _1 = \sigma _2$ in the following, and that ${{\hat{\sigma }}}$ is a sufficiently good estimate of this common variance.

The likelihood of the difference parameter $\Delta _\mu = \mu _1 - \mu _2$ on a dataset translates to the likelihood on the values of *d* from Theorem 1. If we approximate this likelihood by a normal distribution, which it will asymptotically become, we get

36 $$\begin{aligned} \mathbb {V}(d|\Delta _\mu )= & {} \frac{{\hat{\sigma }}^2}{N \mathbb {V}(p)} + \Delta _\mu ^2 \frac{\sum _{i=1}^N (p_i-{\overline{p}})^2 p_i (1-p_i)}{N^2 \mathbb {V}(p)^2} \end{aligned}$$

37 $$\begin{aligned}= & {} \frac{\sum _{i=1}^N (x_i - {\overline{x}})^2}{N \mathbb {V}(p) (N-1)} + \Delta _\mu ^2 \left( \frac{\sum _{i=1}^N (p_i-{\overline{p}})^2 p_i (1-p_i)}{N^2 \mathbb {V}(p)^2} - \frac{{\overline{p}} (1-{\overline{p}})}{(N-1) \mathbb {V}(p)} \right) \end{aligned}$$

38 $$\begin{aligned}= & {} \frac{\sum _{i=1}^N (x_i - {\overline{x}})^2}{N \mathbb {V}(p) (N-1)} + \Delta _\mu ^2 \frac{\sum _{i=1}^N (p_i-{\overline{p}})^2 \left( p_i (1-p_i) - {\overline{p}}(1-{\overline{p}})\right) }{N^2 \mathbb {V}(p)^2} \end{aligned}$$

39 $$\begin{aligned} L(d|\Delta _\mu )= & {} \mathcal {N}_{d, \mathbb {V}(d|\Delta _\mu )} \end{aligned}$$

40 $$\begin{aligned}= & {} \frac{1}{\sqrt{2 \pi \mathbb {V}(d|\Delta _\mu )}} \mathrm {exp}\left( -\frac{1}{2} \frac{(d - \Delta _\mu )^2}{\mathbb {V}(d|\Delta _\mu )}\right) . \end{aligned}$$

Note that the variance term can become negative for large absolute values of $\Delta _\mu$ since the variance in the data is not sufficiently large to justify such a large $\Delta _\mu$. In these cases, the likelihood is zero. In consequence, the likelihood is zero everywhere but in a finite interval around zero.

For a general prior on $\Delta _\mu$, the resulting integral can be solved by numerical integration; for a flat prior on the real numbers, the equation itself is the integrant. For example, to compute the a-posteriori probability that $\Delta _\mu$ is below a threshold *a* (in many instances, zero) under a flat prior would be

$$\begin{aligned} P(\Delta _\mu < a|d, p_1,...,p_N) = \int _{-\infty }^a L(d|\delta _\mu ) d\delta _\mu . \end{aligned}$$

## Practical Computations

In this section, we summarize the most important computations to perform the frequentist test against equal means in two groups where only the probabilities of group memberships are known.

Again, we denote each data vector for participant $i = 1...N$ as $(x_i, p_i)$, where $x_i$ is the target value and $p_i$ the probability that the participant is in Group 1. We assume that the probabilities are known and that within each group, the variable is normally distributed and independent of the probability. The normality assumption is only required for the *t*-distribution of the test statistic, the descriptive statistics are correct for any distribution with finite variance. We then compute

41 $$\begin{aligned} {\overline{x}}= & {} \frac{1}{N} \sum _{i=1}^N x_i \end{aligned}$$

42 $$\begin{aligned} {\overline{p}}= & {} \frac{1}{N} \sum _{i=1}^N p_i \end{aligned}$$

43 $$\begin{aligned} z= & {} \sum _{i=1}^N (p_i - {\overline{p}}) x_i \end{aligned}$$

44 $$\begin{aligned} d= & {} \frac{z}{N \mathbb {V}(p)} \end{aligned}$$

45 $$\begin{aligned} {\hat{\sigma }}^2= & {} \frac{1}{N-2} \left( \left( \sum _{i=1}^N (x_i - {\overline{x}})^2\right) - N {\overline{p}} (1-{\overline{p}}) d^2 \right) \end{aligned}$$

46 $$\begin{aligned} \mathbb {V}(p)= & {} \frac{1}{N} \sum _{i=1}^N (p_i - {\overline{p}})^2 \end{aligned}$$

47 $$\begin{aligned} t= & {} d \cdot \frac{\sqrt{N \mathbb {V}(p)}}{{{\hat{\sigma }}}}. \end{aligned}$$

The following values are not needed for the test, but provide statistics for the means of both groups, the standard deviation of both groups, and a 95 % confidence interval for the difference *d* in the group means. Note that while *d* is asymptotically normally distributed, for lower *N* the standard error does depend on the actual mean difference.

48 $$\begin{aligned} {\hat{\mu }}_1= & {} {\overline{x}} + d (1-{\overline{p}}) \end{aligned}$$

49 $$\begin{aligned} {\hat{\mu }}_2= & {} {\overline{x}} - d {\overline{p}} \end{aligned}$$

50 $$\begin{aligned} z_2= & {} \sum _{i=1}^N (p_i - {\overline{p}}) x_i^2 \end{aligned}$$

51 $$\begin{aligned} {\hat{\sigma }}_1^2= & {} \left( \frac{\sum _{i=1}^N x_i^2}{N} - \frac{(1-{\overline{p}}) z_2}{N \mathbb {V}(p)}\right) - {\hat{\mu }}_1^2 \end{aligned}$$

52 $$\begin{aligned} {\hat{\sigma }}_2^2= & {} \left( \frac{\sum _{i=1}^N x_i^2}{N} - \frac{{\overline{p}} z_2}{N \mathbb {V}(p)}\right) - {\hat{\mu }}_2^2 \end{aligned}$$

53 $$\begin{aligned} stderr= & {} \sqrt{\frac{ \sum _{i=1}^N (p_i - {\overline{p}})^2 \left[ p_i (1-p_i) d^2 + p_i \sigma _1^2 + (1-p_i) \sigma _2^2\right] }{N^2 \mathbb {V}(p)^2}} \end{aligned}$$

54 $$\begin{aligned} CI_{95\%}(d)= & {} [d - 1.97 stderr, d + 1.97 stderr]. \end{aligned}$$

To conclude the test, the researcher finally computes the probability *p* that a *t*-distribution with $N-1$ degrees of freedom provides a value equal to or above *t*, or an absolute value above *t* for a two-sided test. For a frequentist test, *p* can then be compared to a a-priori fixed $\alpha$ value.

For a Bayesian test, the likelihood for parameter values (for the mean difference) close to the estimated mean difference can be assumed to follow a normal distribution by the central limit theorem. Using this distribution and a desired prior on the mean difference yields a good approximation of the posterior. For smaller *N*, the likelihood for any parameter value can be approximated more precisely by using a normal distribution with standard deviation using the *stderr* term above for the given mean difference. For very small *N*, the likelihood is a mixture of Binomial distributions; however, for usual sample sizes the normal approximation should be sufficient.

### Remarks

Note that the technique assumes that probabilities for group memberships are known. Violations of this assumption may lead to biased estimates. For example, if a correct probability for group membership is “watered down” by proportionally reducing $p-{\overline{p}}$ for all participants (e.g., when replacing 0 and 1 for group membership by probabilities 0.1 and 0.9 on equal-sized groups), the mean estimate increases by this factor. We can see this by reducing $p-{\overline{p}}$ by a factor *c* in the equation for *d*:

$$\begin{aligned} d' = \frac{\sum _{i=1}^n c (p-{\overline{p}}) x_i}{N c^2 \mathbb {V}(p)} = \frac{1}{c} \frac{\sum _{i=1}^n (p-{\overline{p}}) x_i}{N \mathbb {V}(p)} = \frac{1}{c} d. \end{aligned}$$

Note, however, that this does not hold for the test statistic, which does not change in expectation:

$$\begin{aligned} t' = d' \cdot \frac{\sqrt{N c^2 \mathbb {V}(p)}}{{{\hat{\sigma }}}} = t \end{aligned}$$

and analogously for the evidence *d* over its standard error.

## Simulation Study

Three simulation studies were performed to test the uncertain group *t*-test. Firstly, the power of the uncertain group *t*-test to reject the null hypothesis postulating no group differences was tested for multiple effect sizes, i.e., mean differences between the groups. In the second simulation, the $\alpha$ inflation was tested if the probabilities for the group memberships were estimated incorrectly, i.e., placed too close to one for high values, or zero for low values, respectively. In the third simulation, the uncertainty was ignored and not taken into account. The groups were assigned by rounding probabilities <.5 to 0 and >.5 to 1 and compared to the results of the first simulation.

### Data Generation

To generate data in which two groups differ in their mean by a certain effect size, but the group membership is only known as a probability, we performed the following steps. First, we assigned a normal distribution to both groups. The variances of both groups were fixed to one. The mean for Group 1 was fixed to zero, while the mean for Group 2 was set to a number between zero (no effect) to one.

For every participant, we assigned a probability to be in Group 2. This probability was randomly drawn from a uniform distribution between zero and one. The true group membership for each participant was then simulated randomly, with the participant being in Group 2 with the chosen probability, and in Group 1 otherwise. For example, if the probability for a participant was randomly chosen to be 35%, then this participant’s true group was Group 2 with probability 35% and Group 1 with probability 65%. Then, the dependent variable was chosen dependent on the true group: If a participant is in Group 1, his *x* value was chosen from a normal distribution with mean zero, and with the mean from Group 2 otherwise. Finally, the true group membership was removed from the simulated dataset, so that only the probability to be in Group 2 and the dependent variable remained. In this vein, data for up to $N=1000$ participants was simulated to create the full dataset for each trial. In this case, one trial equals a significance test for each drawn sample, and therefore the computation of effect, p value, and effect size. A total of 1,000 trials were simulated for each effect size.

The first simulation assumed that the group membership is known as a probability. The probability could either be provided/given by an expert or a classifier. If the provided probability is inaccurate one assumption is violated. To analyze the outcome of the test with biased data, a different simulation was computed.

For the second simulation in which the dataset was assume to violate the assumption of knowing the probabilities, all probability values were exaggerated toward 0 or 1, respectively. When $p_{i}<.5$, the $p_i$ value was divided by 2. If $p_{i}>.5$ the new $p_i$ value was calculated by $\frac{1 - p_{i}}{2} + p_{i}$. Therefore, the $p_{i}$s $<.5$ were moved closer to zero and the p$_{i}$s $>.5$ were moved closer to one.

The final simulation simply ignored the uncertainty and is similar to our second simulation. Contrary to the procedure in our proposed method, where the group membership probabilities are used, in this instance groups were assigned by rounding probabilities to 0 and 1. For probabilities $< .5$, the probability was changed to 0, while all probabilities $> .5$ were changed to 1.

### Data Analysis

The simulated data in each trial of the simulations was then analyzed identically, following the instructions in the Practical Computation Section. Group variances are assumed to be equal in the given procedure. We computed the estimated group difference *d* and the corresponding *t*-value for a test against zero group difference in each trial. We then counted how many percent of the trials in each condition were significant at an $\alpha$ level of 5%. The ratio of significant trials provides the true $\alpha$ level (for no effect) or the power (for nonzero effects), respectively.

The R script for the data generation and analysis can be found in the online supplemental material. The function provided there has three parameters, the effect size, the total number of trials, and the number of participants *N*.

### Simulation Results

**Fig. 1:**
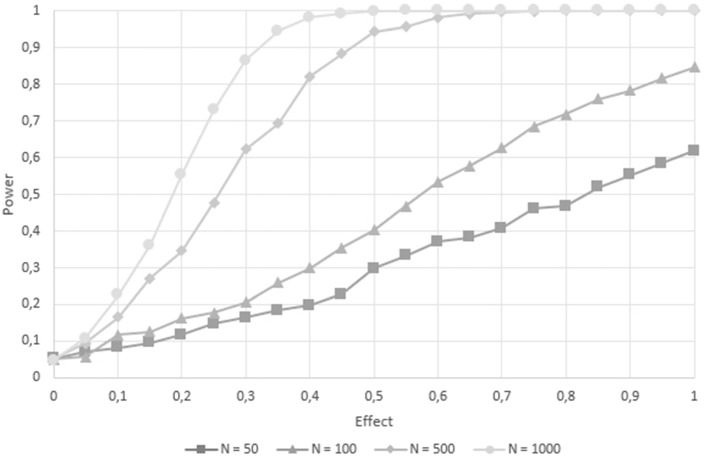
Power curves mapping effect size (*x*-axis) against power (*y*-axis) for sample sizes $N=50$, $N=100$, $N=500$, and $N=1,000$, respectively.

We investigated the power and type I error based on 21 different effect sizes (from zero to one in steps of 0.05) crossed by four sample sizes of $N=50, 100, 500$, and 1000. Figure 1 plots the measurement occasions for each sample size.

For zero true effect, i.e., when the data was simulated with no group differences, 4.3% of the 1000 trials were significant for $N=50$, 5.4% for $N=100$, and 4.2% for $N=500$. The standard error for 1000 trials at a true value of 5% is .69%, that is, the 95% confidence intervals for all three conditions include the nominal $\alpha$ level of 5%. To increase precision, we simulated 10,000 iterations for the largest sample size condition of $N=1000$, resulting in 4.72% ($CI_{95\%}$ = [4.29% ; 5.15%]) significant trials. Note, however, that for even lower *N*, we start to get an $\alpha$ inflation because of the usage of a normal distribution of *z*. For example, we additionally simulated 10.000 trials with $N=20$, which resulted in 7.5% ($CI_{95\%}$ = [7.07% ; 7.93% ]) of the cases being significant.

For effect sizes greater than zero, power increases in all conditions with increasing effect size. With a sample size of $N=50$, the power exceeded 80% as soon as the effect size was approximately 1.6 and was greater than .95 from an effect of three or higher. In the second condition, the sample size of $N = 100$ required an effect of 0.95 to reach a power of 80%, and reached values above 95% for effect sizes above 1.4. When sample size increased to 500, the power exceeded 80% at an effect size of 0.40 and reached 95% when the effect was approximately 0.55 or larger. With the largest sample size of 1000 participants, a power of 80% was reached with an effect of 0.3, and exceeded 95% with an effect size of 0.40.

For power values approximately at 100%, a sample size of 50 required an effect of 5, while the next larger sample size of 100 requires an effect of 2. For $N=500$ and $N=1000$ participants, power is close to perfect for true effects of 0.75 and 0.5, respectively, when unbiased probabilities are considered.

These results show the test power without any violations of assumptions. To explore the test vulnerability to assumption violations a second sample was simulated which included inaccurate estimations of $p_{i}$. More precisely, the estimated $p_{i}$ tend more toward the upper and lower extremes due to false assumptions like overestimation. This second, bias-induced simulation dataset was also analyzed.

**Fig. 2:**
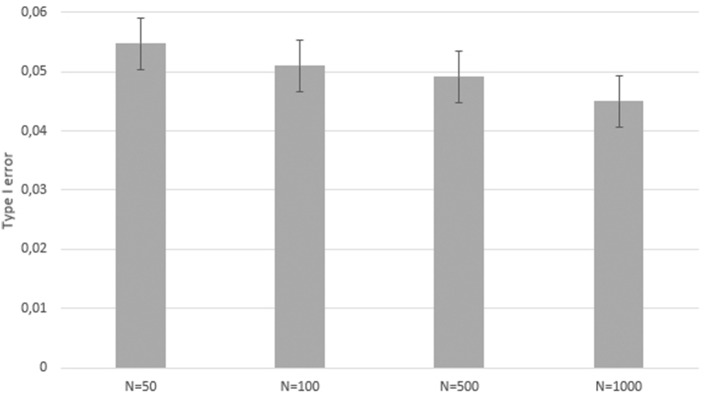
Type I error with biased data for sample sizes $N=50$, $N=100$, $N=500$, and $N=1,000$.

The study shows that there is no significant $\alpha$ inflation if the uncertain group *t*-test assumptions are violated. Figure 2 plots the type I error for each sample size. With a sample size of $N = 50$, the test shows the probability of a type I error of 5.47% within 10,000 iterations and effect = 0. The type I error is similar for a sample size of $N = 100$. During 10,000 iterations, the test provided a significant result for 510 iterations with effect set to 0. For $N = 500$, the type I error is .049, for $N = 1000$ the type I error is .045. Both sample sizes were simulated with 10,000 iterations and an effect of 0.

**Fig. 3:**
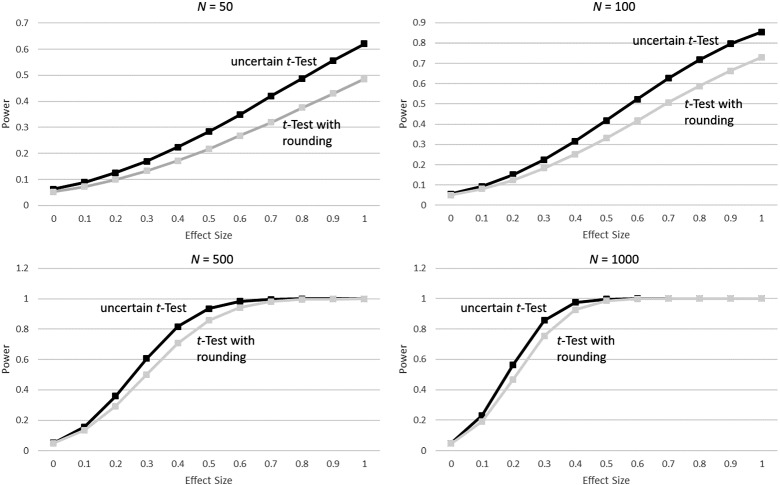
Comparison of power when using the uncertain group *t*-test vs. a classical *t*-test with rounding probabilities to either zero or one, for sample sizes of $N=50$, $N=100$, $N=500$, and $N=1,000$ with different effect sizes on the *x*-axis.

The previously shown simulations take uncertainty into account. To compare this to an ad hoc solution that rounds all probabilities either to zero or to one and then uses a classical *t*-test without uncertainty, a third simulation was set up. Figure 3 visualizes the difference between these two approaches. The results show that for effect sizes greater than zero, power is always consistently lower for the classical *t*-test than for the uncertain group *t*-test in this situation. With a sample size of $N=50$, the power stays below 40% throughout, up to more than a 10% loss compared to the uncertain *t*-test. For conditions with higher *N* and consequently higher power, the difference is maximal for medium ranges of effect. This would be expected as power approaches 100% for both if the effect is larger, and the nominal alpha level of 5% as the effect size approaches zero.

To investigate how the entropy of the distribution of probabilities effects the power, the same simulation was repeated with different entropies of the probability to be in either group instead of different effect sizes. Probabilities were chosen from a Beta distribution with equal parameters $\alpha = \beta$ ranging from 1 (a uniform distribution) to zero (the degenerate case without uncertainty). Effect size was fixed to 0.2 for this simulation.

**Fig. 4:**
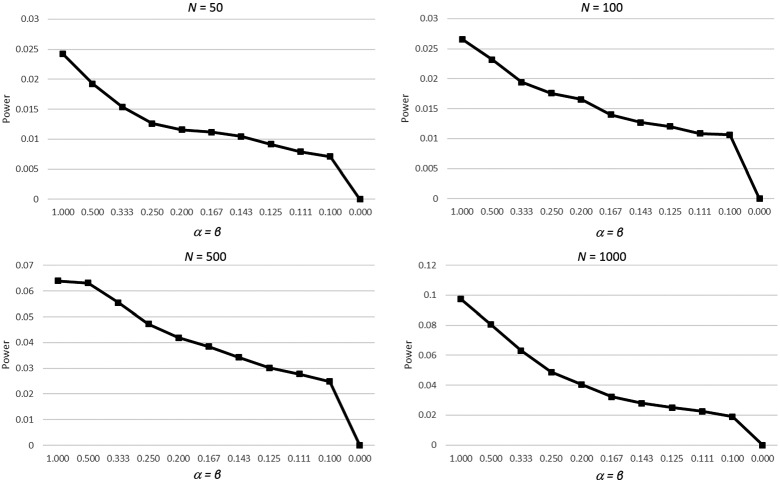
Power difference between the uncertain group *t*-test vs. a classical *t*-test with rounding probabilities to either zero or one, for sample sizes of $N=50$, $N=100$, $N=500$, and $N=1,000$, for different entropies (represented by the parameters of a Beta distribution) on the *x*-axis, in steps of $\frac{1}{x}$ with x ranging from 1 (uniform distribution) to 10 (distribution with high densities close to zero and one)

Figure 4 shows the result as difference of power with a classical *t*-test, rounding all probabilities to zero or one, to the uncertain group *t*-test. For the degenerate case (at the right end of the plots), both tests were identical. For higher entropy (values further left), the gain was higher the more entropy was present in the distribution. The qualitative pattern is not different for different sample sizes.

## Example of Use

We used the openly available data of the teacher questionnaire in the PISA 2018 study (OECD 2018) to conduct two example analyses using the here presented method. The first example shows a case where missing data is substituted for a probability. The second example depicts a situation where the grouping variable had not been assessed. The R script for both can be found in the online supplemental material.

### First Example: Missings in Grouping Variable

The chosen research question is whether teachers born in the country they are currently teaching in differ in the satisfaction with their job from those born in a different country. The job satisfaction is included in the dataset as a weighted likelihood estimation (Warm 1989), ranging from $-3.22$ to 1.62 with an average of 0.09 ($SD = 1.01$). Country of birth was assessed as a dichotomous variable and coded as 0—country of test, and 1—other. A total of 62,065 teachers can be included in the mean comparison with a classical *t*-test, of whom 14.05% were born in a country different from the one they currently taught in. Using the traditional test, the comparison does not reach significance, $t(11789) = 1.81, p = .071$. Additional 27,621 teachers were assessed for the job satisfaction, but omitted the item concerning their country of birth. As a preparation for the here described method, these missings were substituted for the probability in the total sample to be born in a country different from the one they currently taught in, that is .14. The new test statistic was then computed as described under Practical Computations. The results suggest that teachers born in the country they taught in were more satisfied with their job than those born in a different country, $t(89685) = 4.10, p < .001$. Note that this analysis likely constitutes a conservative test. A teacher with a migration background who faces racism is probably less likely to acknowledge this background, while also being less satisfied with their job. This means that the probability of a migration background might have been underestimated for teachers with missing values, which in line means that the lower job satisfaction of these teachers was also rather assigned to teachers without a migration background.

### Second Example: Missing Grouping Variable

In the second example, we want to test whether teachers who share a household with at least one child are more satisfied with the teaching profession than teachers who do not share a household with a child in Germany. The satisfaction with the teaching profession was included in the dataset as a weighted likelihood estimation, ranging from $-2.82$ to 1.61 with an average of 0.41 ($SD = 0.94, N = 4,861$). It was not assessed whether a child lived in the household of the teacher. Age and sex of each teacher were used to assign a probability for a child in the household of every teacher. The probabilities are based on the prevalence in Germany (Destatis 2019). The here presented test was then computed using the probabilities as the grouping variable. The results indicate that teachers in Germany without a child in their household are less satisfied with the teaching profession than those with at least one child in their household, $t(4787) = 2.08, p = .038$.

## Discussion

Psychologists are interested in group differences and commonly use the *t*-test to explore such mean differences in groups. The *t*-test requires an independent grouping variable and one dependent outcome variable. Unfortunately, missing data is a well-known issue in research. Missings in the grouping variable would at least lead to a reduced power in the *t*-test, or in the extreme case prevent its usage overall. The current article provided a method to analyze data with uncertain group membership. Data for this method have a probability of group membership for each participant in addition to the dependent variable. The test assumes that the dependent variable is normally distributed and that the probability values are part of the data and known, and in particular independent of the target variable within groups. The simulation study has shown that the uncertain group *t*-test is correct, has considerable power, and is robust at least to mild assumption violations.

The results reveal that the provided method is a viable solution to the issue of missing or uncertain grouping variables. For zero true effect, 5.17% of the 10,000 trials were significant for *N*=50, 5.04% for *N*=100, and 4.83% for *N*=500. The largest sample size condition of *N*=1000 had 472 (4.72%) significant trials, which shows that the upper endpoints of the $CI_{95\%}$ of the type I error for all conditions with sample sizes from $N=50$ up to $N=1000$ exceed the 5%. Nonetheless, the lower endpoints are all within the 5% $\alpha$ error. This means the uncertain group *t*-test could be vulnerable to type I errors for smaller sample sizes ($\le$N$=100$), but seems to be reliable for larger sample sizes ($\ge$N$=500$).

The uncertain group *t*-test can be applied in multiple situations. Two examples for its use are provided. The first example depicts a use case where missings in the grouping variable occur. The classical *t*-test would have yielded a nonsignificant result, while our proposed test could find the mean differences in the outcome variable through the additional use of participants with missings in the grouping variable. The second example depicts how the proposed test could be used to investigate mean differences when the grouping variable had not been collected in the data. The population prevalence for different age and sex groups has been used to estimate the probability of group membership in this case. This allowed for the investigation of a hypothesis which could otherwise not have been investigated given the collected dataset. Yet, since this method is designed for comparing two groups, it is important to realize that gender is not a dichotomous variable. If for example some participants for which the gender is not explicitly given may self-identify as neither male nor female, the comparison is really a comparison of at least three groups, and the method no longer applicable.

The method has its limitations where the assumptions of the test are violated. In addition to the classical *t*-test assumptions (normal distribution and variance homogeneity), these include that the group probabilities are correct and in particular not dependent to the target within group, or exaggerated or understated. While the conditional independence is usually a valid assumption in many cases (whenever the information underlying the creation of the probabilities in itself is not dependent on the target variable), the second source of probability bias may arguably occur more often, for example, if human raters are too confident or not confident enough in their estimation of group membership. For this assumption violation, the second simulation took biased data like this into account. The probability to be in Group 2 was moved closer to 1 for $p_{i}$s $>.5$ and closer to 0 for $p_{i}$s $<.5$. The simulation outcome shows that the test has no strong $\alpha$ inflation even though the probabilities are biased. For zero true effect, 5.47% of the 10,000 trials were significant for $N=50$, 5.1% for $N=100$, 4.91% for $N=500$, and 4.5% for $N=1000$. The type I error for each sample size with biased data is within the respective $CI_{95\%}$ of the type I error without biased data. The effect of this assumption violation, therefore, seems to be negligible overall. Note in particular that for the highest *N* of 1000, the test is even very slightly conservative, with a type I error below the nominal $\alpha$ level.

The third simulation was computed to compare the benefit of taking uncertainty into account. The proposed method does not simply split the participants into two groups based on the probability to be in Group 1 or Group 2. This procedure leads to overconfidence and high power even for small effect sizes. The type I error for zero true effect revealed a $\alpha$ inflation and demonstrates to which degree the uncertainty is worth properly accounting for.

Further studies should take different cases of biased data into consideration. The probability to be in Group 1, for example, could be closer to the mean according to the error of central tendency. This can occur if the observer wants to make no mistakes and tries to give rather conservative probabilities. Another possible reason for central tendency is a lack of motivation and consequently attention. In addition to that, the data can be biased if the observer tends to assign more participants to the group of the observer. Continuing the given example of place of residence of miners as the grouping variable, a foreman living next to the mine could tend to give higher probabilities for participants to also live next to the mine, while the probability to live further away tends more toward .5. The example works vice versa for foremen not living next to the mines. In order to further explore the vulnerability of the uncertain group *t*-test to assumption violations different studies should take more assumption violations into account and evaluate the power of the uncertain group *t*-test with biased data.

In conclusion, the method provided here is capable to test for mean group differences even if the group membership is, for some or all participants, unknown, as long as it is replaced by a group membership probability. This may open possibilities to investigate situations which so far have been very difficult or even impossible to assess since group membership is difficult to assess (e.g., a complex biological marker), very sensitive and therefore not or possibly not truthfully given (e.g., drug addiction or criminal background), is unavailable in a pre-existing dataset (e.g., when performing a secondary data analysis on large-scale longitudinal studies), or is just partially missing due to practical data collection issues. Further research would be needed for cases where the probability of group membership is strongly biased, although simulations provide some evidence that the method shows at least some robustness to assumption violations.
